# Comparison of Survival Outcomes Among Patients With Breast Cancer With Distant vs Ipsilateral Supraclavicular Lymph Node Metastases

**DOI:** 10.1001/jamanetworkopen.2021.1809

**Published:** 2021-03-16

**Authors:** Hong Pan, Hui Wang, Mengjia Qian, Xinrui Mao, Guojian Shi, Ge Ma, Muxin Yu, Hui Xie, Lijun Ling, Qiang Ding, Kai Zhang, Shui Wang, Wenbin Zhou

**Affiliations:** 1 Department of Breast Surgery, The First Affiliated Hospital with Nanjing Medical University, Nanjing, China; 2 Jiangsu Key Lab of Cancer Biomarkers, Prevention and Treatment, Jiangsu Collaborative Innovation Center for Cancer Personalized Medicine, School of Public Health, Nanjing Medical University, Nanjing, China; 3 Department of Thyroid and Breast Surgery, The Second Affiliated Hospital of Soochow University, Suzhou, China; 4 Department of Thyroid and Breast Surgery, The Second People’s Hospital of Kunshan, Suzhou, China; 5 Pancreatic Center & Department of General Surgery, The First Affiliated Hospital with Nanjing Medical University, Nanjing, Jiangsu, China; 6 Pancreas Institute of Nanjing Medical University, Nanjing, Jiangsu, China

## Abstract

**Importance:**

There is a lack of studies exploring whether the survival of patients with distant lymph node metastases (DLNM) is different from that of patients with ipsilateral supraclavicular lymph node metastases (ISLM) and other stage IV breast cancer.

**Objective:**

To assess the survival of patients with DLNM from breast cancer vs ISLM and other stage IV breast cancer.

**Design, Setting, and Participants:**

This cohort study included 2033 patients diagnosed with breast cancer between January 1, 2010, and December 31, 2014, from the Surveillance, Epidemiology and End Results registries database. Three groups of patients were included: (1) patients with ISLM without any distant metastasis, (2) patients with DLNM, and (3) patients with distant metastases (DLNM excluded). Patients younger than 18 years or older than 100 years were excluded. The data were analyzed in February 2020.

**Exposures:**

Surgery for primary tumor, surgery for distant lymph nodes, and radiotherapy.

**Main Outcomes and Measures:**

Overall survival (OS) and breast cancer–specific survival (BCSS).

**Results:**

Of the 2033 women (mean [SD] age, 62.03 [14.62] years [range, 23.00-99.00 years]; 1510 White participants [74.3%]) with breast cancer included in the study, 346 patients (17.0%) had DLNM, 212 (10.4%) had ISLM, and 1475 (72.6%) had distant metastases (DLNM excluded). The 3-year BCSS rates were 63.24% for ISLM, 64.54% for DLNM, and 41.20% for distant metastases. The 3-year OS rates were 53.46% for ISLM, 62.67% for DLNM, and 38.21% for distant metastases. Compared with patients with ISLM, patients with DLNM showed similar BCSS (hazard ratio [HR], 0.81; 95% CI, 0.52-1.25; *P* = .34) and OS (HR, 0.73; 95% CI, 0.51-1.05; *P* = .09), whereas patients with distant metastases showed significantly poorer BCSS (HR, 1.99; 95% CI, 1.43-2.78; *P* < .001) and OS (HR, 1.79; 95% CI, 1.35-2.38; *P* < .001). Of the 346 patients with DLNM, primary surgery (HR, 0.21; 95% CI, 0.12-0.39; *P* < .001) and radiotherapy (HR, 0.46; 95% CI, 0.25-0.87; *P* = .02) were significantly associated with improved OS.

**Conclusions and Relevance:**

The results of this cohort study suggest that DLNM of breast cancer, with similar survival to N3c disease (indicating metastases to the ipsilateral supraclavicular lymph nodes), might be a regional disease, and reassessment of the role of lymph node metastases in breast cancer may be necessary. Given these findings, aggressive locoregional therapies for this disease are recommended, although future studies are still needed to confirm these results.

## Key Points

**Question:**

Is the survival of patients with distant lymph node metastases (DLNM) different from that in patients with ipsilateral supraclavicular lymph node metastases (ISLM) and other stage IV breast cancer?

**Findings:**

This population-based cohort study of 2033 patients with breast cancer found that compared with patients with ISLM, patients with distant metastases had significantly poorer breast cancer–specific survival and overall survival, whereas patients with DLNM had similar breast cancer–specific survival and overall survival. Primary surgery and radiotherapy were significantly associated with improved overall survival for patients with DLNM.

**Meaning:**

These findings suggest that DLNM of breast cancer may be a regional disease, which could benefit from locoregional therapies.

## Teaser

This cohort study evaluates the association of distant vs ipsilateral supraclavicular lymph node metastases with survival among patients with breast cancer.

## Introduction

Breast cancer is a common malignant disease worldwide. The incidence of synchronous distant metastases in patients with newly diagnosed breast cancer is approximately 3% to 8%.^1,2,3^ Unfortunately, stage IV breast cancer is an incurable disease with a poor prognosis. Patients with central nervous system or liver metastases show poorer survival than patients with other metastasis sites, including bone, the lungs, and distant lymph nodes.^4,5^ Systemic therapy is the main treatment for stage IV breast cancer to prolong patient survival and palliate the symptoms. However, with advances in systemic therapies, highly selected stage IV breast cancer (eg, patients with bone or distant lymph node metastases [DLNM]) may be potentially curable, especially for limited metastatic burden and favorable site cases.^6,7,8^ Understanding the characteristics of breast cancer metastasis is necessary to select the appropriate patients for precise and aggressive therapy.

Organ-specific clinical metastases are derived from cells that escape from the primary cancer and metastasize to and grow within the liver, lungs, lymph nodes, or other organs. Previous studies suggest that regional lymph node metastases themselves are nonlethal but can be indicative of distant organ metastases and prognosis.^7,8^ Axillary lymph nodes, the infraclavicular nodes, the supraclavicular nodes, and distant nodes (cervical, contralateral axillary or internal mammary, transverse cervical, and others) are part of a continuum in the lymph node drainage system of the breast,^9^ and they are separated according to arbitrary anatomic boundaries and prognostic indications.^9,10^ However, there is a robust ongoing debate about the role of lymph node metastases in further progression of disease.^7,8,11^ Some studies suggest that lymph node metastases have the potential to seed distant organs, and lymph node blood vessels are a gateway for the transit of nodal tumor cells into the systemic circulation.^11,12,13^ However, other studies contend that localized lymph node metastases are clinically inconsequential.^8,11,14^ Further exploration is needed to demonstrate whether there is any difference in prognosis and treatment between patients with lymph node metastasis and other types of stage IV breast cancer.

Patients with ipsilateral supraclavicular lymph node metastases (ISLM) from breast cancer, included in the stage IV category before 2002^9^ according to the American Joint Committee on Cancer (AJCC) classification, develop distant metastases within 1 year of detection of this disease.^15^ These patients are treated with radiotherapy alone or in combination with surgical resection as the previous standard of care.^16^ With advances in chemotherapy and endocrine therapy, patients with ISLM show similar survival to those with stage IIIB disease but a much better survival than those with stage IV disease.^9^ Therefore, this disease was staged as N3c (indicating metastases to the ipsilateral supraclavicular lymph nodes) in the sixth edition of the AJCC classification. As is known, DLNM, including to cervical, contralateral axillary or internal mammary, transverse cervical, and other nodes, are still considered to be stage IV diseases. As lymph system metastasis, DLNM are theoretically anatomically distant compared with ISLM. Whether or not patients with DLNM show poorer survival than patients with ISLM or similar survival is unknown.

With advances in modern systemic therapies,^6^ relatively favorable survival of patients with lymph node metastases,^7,8^ and improved understanding of the role of lymph nodes in metastasis,^11,12^ the clinical role of DLNM in breast cancer should be assessed. The purpose of the current study was to assess the survival of patients with breast cancer with DLNM vs ISLM and other distant metastases in a large, population-based cohort study.

## Methods

This cohort study included patients diagnosed with breast cancer between January 1, 2010, and December 31, 2014. With approval from the review board of the First Affiliated Hospital with Nanjing Medical University, the study was deemed exempt from research ethics board approval and informed consent because the study participants were ascertained through the Surveillance, Epidemiology, and End Results (SEER) database. The present study followed the Strengthening the Reporting of Observational Studies in Epidemiology (STROBE) reporting guideline.

### Data Source and Study Sample

The data applied in the current study were from the National Cancer Institute’s SEER program, which began its data collection on January 1, 1973 and is ongoing (http://www.seer.cancer.gov). Because of a lack of *ERBB2* (formerly *HER2* or *HER2/neu*) status before 2010, only patients diagnosed with breast cancer from 2010 to 2014 were included. Patients younger than 18 years or older than 100 years were excluded. Three groups of patients were included in the analysis: (1) patients with ISLM with any tumor (T) and axillary node (N) stage without any distant metastasis, (2) patients with only DLNM and any T and N stage, and (3) patients with distant metastases (DLNM excluded) and any T and N stage. DLNM included cervical, contralateral or bilateral axillary or internal mammary, transverse cervical, or other distant node metastases from the SEER database (eTable 1 in the Supplement).

### Patient and Tumor-Related Variables

Patient characteristics included age at diagnosis, race/ethnicity, and year of diagnosis. Tumor-related variables included histology; tumor grade; T, N, and metastasis (M) classification from the seventh edition of the AJCC; estrogen receptor (ER) status; progesterone receptor (PR) status; *ERBB2* status; and molecular subtype (directly from the database). Hormone-receptor positive (HR+) was defined as ER and/or PR positive, and hormone-receptor negative (HR–) was defined as ER and PR negative.

### Treatment-Related Variables

Surgery for the primary tumor was determined from SEER claims (RX Summ—Surg Prim Site, codes 0 as no primary surgery and 0-98 as primary surgery), and surgery for distant lymph nodes was determined using SEER claims (RX Summ—Surg Oth Reg/Dis, codes 0 as no distant surgery and 3 as nonprimary surgical procedure to distant lymph nodes). Chemotherapy was determined from SEER claims (Chemotherapy Recode [Yes, No/Unk], codes 0 as No or Unknown and 1 as yes). Treatment with radiation was determined using SEER claims (Radiation Recode, codes 0 and 7 as no radiotherapy and codes 1-6 as radiotherapy). However, endocrine therapy and anti-*ERBB2* therapy were not reported in the SEER database.

### Clinical Outcome

The primary outcome of the current study was overall survival (OS), and the secondary outcome was breast cancer–specific survival (BCSS). Overall survival was measured as the time from breast cancer diagnosis to the date of death from any cause (including breast cancer) or the date of last follow-up (Vital Status Recode). Breast cancer–specific survival was defined as the time from the date of breast cancer diagnosis until death caused by breast cancer or the date of last follow-up (SEER cause-specific death classification). We only included patients diagnosed with breast cancer from 2010 to 2014 because of a lack of *ERBB2* status before 2010. Follow-up information was retrieved from the SEER database, and the end date for the follow-up was December 31, 2014.

### Statistical Analysis

Medians, percentiles, and ranges were analyzed for each continuous variable. Unadjusted associations of variables among the 3 groups were assessed using the Pearson χ^2^ test. For survival analysis, the Kaplan-Meier method was applied for each variable in the univariate analysis. The Cox proportional hazards model with independent variables was applied for the multivariate analysis. Independent variables found to have a *P* < .05 in the univariate analysis were further investigated in the multivariate analysis. For subgroup analyses, the Bonferroni correction was applied, and a Bonferroni adjusted *P* value of significance of 0.01 was established. This was done to circumvent the increased chance of a type I error with multiple comparisons. Propensity score matching was performed to further elucidate the characteristics of the patients. The DLNM cases were 1:1 matched with the ISLM cases. Propensity scores were calculated using a multivariable logistic regression model based on factors that could affect the survival of patients with breast cancer, including age, grade, subtype, and histology. All statistical analyses were performed in February 2020 using Stata software, version 11.0 (StataCorp), and a significant difference was concluded for *P* < .05. All *P* values were 2-sided.

## Results

### Baseline Characteristics

Of the 2033 women (mean [SD] age, 62.03 [14.62] years [range, 23.00-99.00 years]; 1510 White participants [74.3%]) with breast cancer included in the study, 346 patients (17.0%) had DLNM, 212 (10.4%) had ISLM, and 1475 (72.6%) had distant metastases (DLNM excluded) (Table 1). Among the 346 patients with DLNM, the median age (range) was 60.50 (18.00-100.00) years, 236 patients (68.2%) were diagnosed with invasive ductal carcinoma (IDC), 170 patients (49.1%) had poorly differentiated or undifferentiated cancer 151 patients (43.6%) had HR+/*ERBB2–* subtype, and 70 patients (20.2%) had triple-negative breast cancer. The baseline characteristics of the 3 groups are shown in Table 1.

**Table 1.** Basic Characteristics of All Included Patients

Variables	No. (%)			*P* value
	ISLM (n = 212)	DLNM (n = 346)	Distant metastases (n = 1475)	
Age, y				<.001
≤50	66 (31.1)	71 (20.5)	273 (18.5)	
>50	146 (68.9)	275 (79.5)	1202 (81.5)	
Race/ethnicity				.95
White	156 (73.6)	253 (73.1)	1101 (74.6)	
Black	35 (16.5)	56 (16.2)	229 (15.5)	
Other^a^	17 (8.0)	34 (9.8)	141 (9.6)	
Unknown	4 (1.9)	3 (0.9)	4 (0.3)	
Histology				.003
IDC	149 (70.3)	236 (68.2)	901 (61.1)	
ILC	15 (7.1)	21 (6.1)	170 (11.5)	
Other	48 (22.6)	89 (25.7)	404 (27.4)	
Grade				<.001
Well differentiated	7 (3.3)	17 (4.9)	100 (6.8)	
Moderately differentiated	44 (20.8)	98 (28.3)	454 (30.8)	
Poorly differentiated or undifferentiated	125 (59.0)	170 (49.1)	516 (35.0)	
Unknown	36 (17.0)	61 (17.6)	405 (27.5)	
Subtype				<.001
HR+/*ERBB2*–^b^	94 (44.3)	151 (43.6)	723 (49.0)	
HR+/*ERBB2*+	24 (11.3)	59 (17.1)	184 (12.5)	
HR–/*ERBB2*+	25 (11.8)	39 (11.3)	128 (8.7)	
HR–/*ERBB2*–	56 (26.4)	70 (20.2)	167 (11.3)	
Unknown	13 (6.1)	27 (7.8)	273 (18.5)	

Abbreviations: DLNM, distant lymph node metastases; HR–, hormone-receptor negative; HR+, hormone-receptor positive; IDC, invasive ductal carcinoma; ILC, invasive lobular carcinoma; ISLM, ipsilateral supraclavicular lymph node metastases.

^a^ Other indicates American Indian, Alaskan native, Asian/Pacific Islander, or unknown.

^b^ *ERBB2* (formerly *HER2* or *HER2/neu*).

### Survival

**Figure 1.**
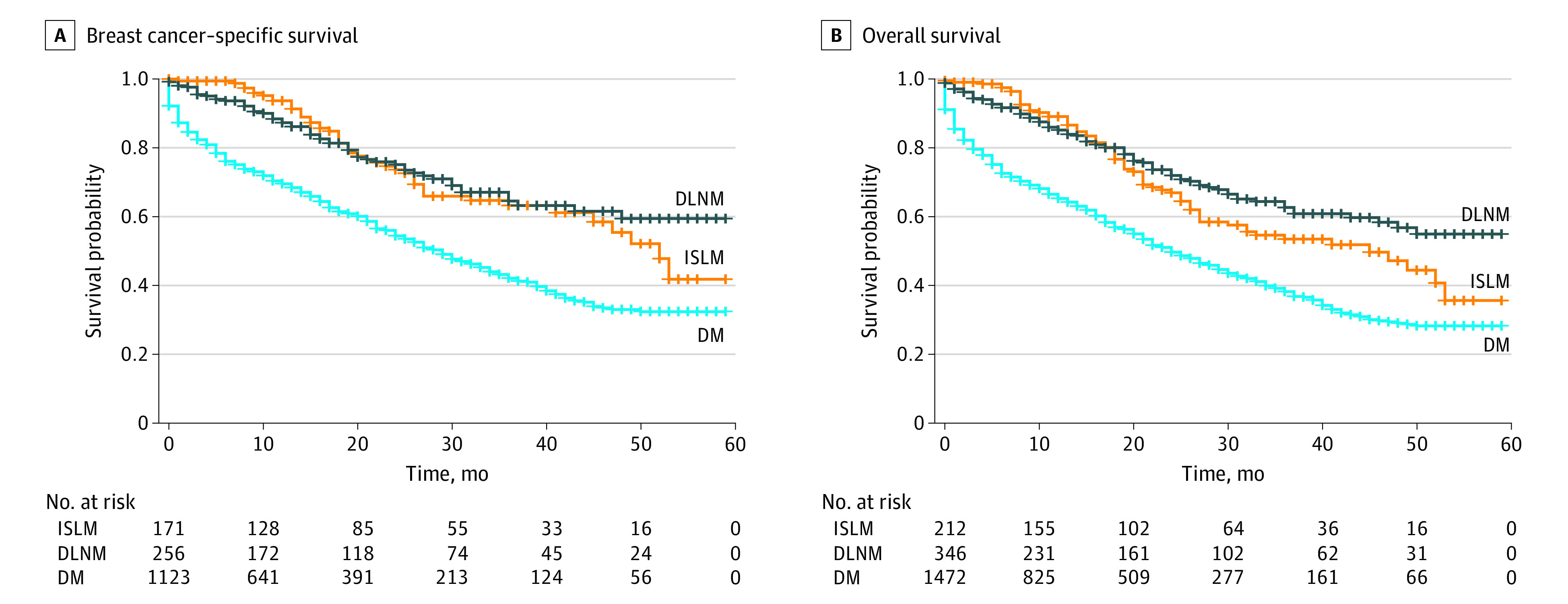
Kaplan-Meier Survival Curves for Patients With Breast Cancer and Distant Lymph Node Metastases (DLNM), Ipsilateral Supraclavicular Lymph Node Metastases (ISLM), and Distant Metastases (DM) Breast cancer–specific survival (A) and overall survival (B) over time.

At a median follow-up of 27 months, the median survival time was 45 (range, 0-59) months for ISLM cases and 24 (range, 0-59) months for distant metastasis cases. The median survival time for patients with DLNM was not reached until the end of follow-up. The 3-year BCSS rates were 63.24% for ISLM, 64.54% for DLNM, and 41.20% for distant metastases. The 3-year OS rates were 53.46% for ISLM, 62.67% for DLNM, and 38.21% for distant metastases. We found (eTable 2 in the Supplement) that patients with DLNM did not show poorer BCSS (hazard ratio [HR] 0.93; 95% CI, 0.64-1.36; *P* = .72) and OS (HR, 0.81; 95% CI, 0.59-1.10; *P* = .17) compared with those with ISLM (Figure 1). However, patients with distant metastases showed significantly poorer BCSS (HR, 2.17; 95% CI, 1.61-2.92; *P* < .001) and OS (HR, 1.90; 95% CI, 1.49-2.42; *P* < .001) than patients with ISLM (Figure 1).

**Figure 2.**
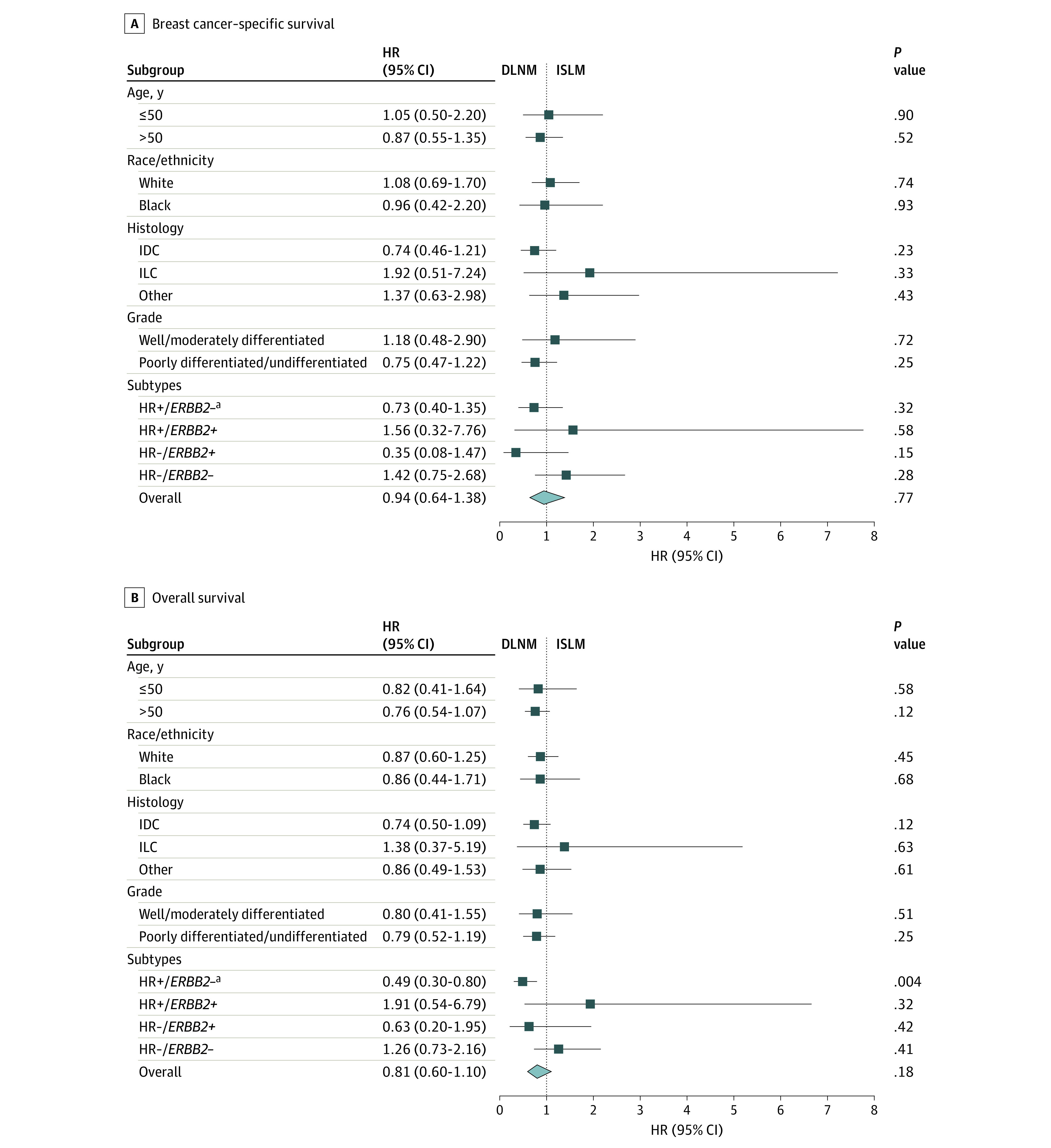
Subgroup Analysis of Breast Cancer–Specific Survival and Overall Survival in Patients With Breast Cancer With Distant Lymph Node Metastases (DLNM) vs Ipsilateral Supraclavicular Lymph Node Metastases (ISLM) Breast cancer–specific survival (A) and overall survival (B) over time. HR indicates hazard ratio; HR−, hormone-receptor negative; HR+, hormone-receptor positive; IDC, invasive ductal carcinoma; ILC, invasive lobular carcinoma. ^a^*ERBB2* (formerly *HER2* or *HER2/neu*).

A subgroup analysis (Figure 2) revealed comparable BCSS and OS between patients with ISLM and those with DLNM across different subgroups, including patients older than 50 years or 50 years or younger; White or Black patients; patients diagnosed with IDC, invasive lobular carcinoma (ILC), or other types; patients with well to moderately differentiated or poorly differentiated to undifferentiated tumor; and patients with different molecular subtypes. By using the Cox proportional hazards models, age, race/ethnicity, histology, molecular subtype, and clinical stage were significantly associated with BCSS and OS (Table 2). Compared with patients with ISLM (Table 2), patients with DLNM showed similar BCSS (HR, 0.81; 95% CI, 0.52-1.25; *P* = .34) and OS (HR, 0.73; 95% CI, 0.51-1.05; *P* = .09), whereas patients with distant metastases showed significantly poorer BCSS (HR, 1.99; 95% CI, 1.43-2.78; *P* < .001) and OS (HR, 1.79; 95% CI, 1.35-2.38; *P* < .001).

**Table 2.** Multivariate Analysis for Breast Cancer–Specific Survival and Overall Survival for All Included Patients

Variable	Breast cancer–specific survival		Overall survival	
	HR (95% CI)	*P* value	HR (95% CI)	*P* value
Age, y				
≤50	1 [Reference]	NA	1 [Reference]	NA
>50	1.61 (1.25-2.07)	<.001	1.72 (1.37-2.14)	<.001
Race/ethnicity				
White	1 [Reference]	NA	1 [Reference]	NA
Black	1.32 (1.02-1.70)	.03	1.30 (1.04-1.61)	.02
Other^a^	1.08 (0.75-1.55)	.68	1.09 (0.81-1.48)	.57
Histology				
IDC	1 [Reference]	NA	1 [Reference]	NA
ILC	1.54 (1.11-2.15)	.01	1.30 (0.98-1.74)	.07
Other	1.47 (1.10-1.96)	.009	1.30 (1.03-1.64)	.03
Grade				
Well differentiated	1 [Reference]	NA	1 [Reference]	NA
Moderately differentiated	1.22 (0.77-1.92)	.40	1.20 (0.84-1.71)	.31
Poorly differentiated or undifferentiated	1.54 (0.96-2.46)	.07	1.42 (0.99-2.05)	.06
Subtype				
HR+/*ERBB2–*^b^	1 [Reference]	NA	1 [Reference]	NA
HR+/*ERBB2+*	1.57 (1.03-2.38)	.04	1.47 (1.02-2.12)	.04
HR–/*ERBB2+*	1.57 (1.13-2.19)	.007	1.47 (1.12-1.94)	.006
HR–/*ERBB2–*	3.09 (2.16-4.43)	<.001	2.95 (2.18-4.00)	<.001
Stage group				
ISLM	1 [Reference]	NA	1 [Reference]	NA
DLNM	0.81 (0.52-1.25)	.34	0.73 (0.51-1.05)	.09
Distant metastases	1.99 (1.43-2.78)	<.001	1.79 (1.35-2.38)	<.001

Abbreviations: DLNM, distant lymph node metastases; HR, hazard ratio; HR–, hormone-receptor negative; HR+, hormone-receptor positive; IDC, invasive ductal carcinoma; ILC, invasive lobular carcinoma; ISLM, ipsilateral supraclavicular lymph node metastases; NA, not available.

^a^ Other indicates American Indian, Alaskan native, Asian/Pacific Islander, or unknown.

^b^ *ERBB2* (formerly *HER2* or *HER2/neu*).

To avoid potential selection bias and imbalance of clinical characteristics, propensity score matching analysis was performed to confirm our results. After 1:1 propensity score matching for age, race/ethnicity, histology, grade, and subtype, 143 pairs of patients were identified in the comparison of the ISLM and DLNM groups (eTable 3 in the Supplement). The results were consistent with multivariable Cox regression. Compared with patients with ISLM, patients with DLNM still showed similar BCSS (HR, 0.83; 95% CI, 0.52-1.33; *P* = .44) and OS (HR, 0.84; 95% CI, 0.54-1.31; *P* = .44).

### Locoregional Treatment of Stage IV Breast Cancer With DLNM

For stage IV breast cancer, systemic therapy is the standard treatment, but the role of locoregional treatment is unclear. The role of locoregional treatment in DLNM was investigated in this study. Of the 346 patients with DLNM, 193 (55.8%) received surgery for the primary tumor, 52 (15.0%) received surgery for distant lymph nodes, and 127 (36.7%) received radiotherapy (eTable 4 in the Supplement).

In univariate log-rank analysis (eTable 5 in the Supplement), axillary node status was not associated with BCSS and OS, and patients with T4 tumors showed poorer OS than those with T1 tumors (HR, 2.39; 95% CI, 1.20-4.76; *P* = .01). Tumor grade was also not associated with BCSS and OS in patients with DLNM. Compared with patients with HR+/*ERBB2*– tumors, patients with triple-negative breast cancer showed poorer BCSS (HR, 2.68; 95% CI, 1.47-4.89; *P* = .001) and OS (HR, 3.11; 95% CI, 1.88-5.15; *P* < .001). Importantly, primary surgery improved both BCSS (HR, 0.22; 95% CI, 0.13-0.39; *P* < .001) and OS (HR, 0.27; 95% CI, 0.18-0.42; *P* < .001), but distant surgery did not. Moreover, radiotherapy was also associated with improved BCSS (HR, 0.32; 95% CI, 0.18-0.59; *P* < .001) and OS (HR, 0.39; 95% CI, 0.24-0.64; *P* < .001).

The roles of locoregional treatment in different molecular subtypes may be different. Subgroup analyses of BCSS and OS revealed a consistent benefit of surgery for primary tumors and radiotherapy for patients with HR+/*ERBB2*– and triple-negative breast cancer. For patients with *ERBB2*+ breast cancer, surgery for primary tumors and radiotherapy were associated with improved OS but not BCSS (eFigure in the Supplement).

By using the Cox proportional hazards models (Table 3), primary surgery was significantly associated with improved BCSS (HR, 0.17; 95% CI, 0.08-0.38; *P* < .001) and OS (HR, 0.21; 95% CI, 0.12-0.39; *P* < .001). Radiotherapy was not associated with significant improvement in BCSS (HR, 0.48; 95% CI, 0.22-1.05; *P* = .07) but was associated with improved OS (HR, 0.46; 95% CI, 0.25-0.87; *P* = .02).

**Table 3.** Multivariate Analysis for Breast Cancer–Specific Survival and Overall Survival for Patients With Distant Lymph Node Metastases

Variable	Breast cancer–specific survival		Overall survival	
	HR (95% CI)	*P* value	HR (95% CI)	*P* value
Histology				
IDC	1 [Reference]	NA	1 [Reference]	NA
ILC	6.30 (1.34-29.74)	.02	2.33 (0.54-10.15)	.26
Other	2.38 (1.16-4.86)	.02	1.50 (0.85-2.66)	.16
T stage				
T1	1 [Reference]	NA	1 [Reference]	NA
T2	3.10 (0.98-9.85)	.06	2.25 (0.94-5.36)	.07
T3	1.34 (0.24-7.54)	.74	2.32 (0.82-6.59)	.11
T4	2.68 (0.92-7.75)	.07	2.93 (1.35-6.36)	.007
Subtype				
HR+/*ERBB2–*^a^	1 [Reference]	NA	1 [Reference]	NA
HR+/*ERBB2+*	0.71 (0.26-1.96)	.51	0.96 (0.45-2.07)	.92
HR–/*ERBB2+*	0.36 (0.08-1.57)	.18	0.49 (0.17-1.41)	.19
HR–/*ERBB2–*	4.91 (2.40-10.06)	<.001	5.10 (2.84-9.16)	<.001
Primary surgery				
No	1 [Reference]	NA	1 [Reference]	NA
Yes	0.17 (0.08-0.38)	<.001	0.21 (0.12-0.39)	<.001
Radiotherapy				
No	1 [Reference]	NA	1 [Reference]	NA
Yes	0.48 (0.22-1.05)	.07	0.46 (0.25-0.87)	.02

Abbreviations: HR, hazard ratio; HR–, hormone-receptor negative; HR+, hormone-receptor positive; IDC, invasive ductal carcinoma; ILC, invasive lobular carcinoma; NA, not available.

^a^ *ERBB2* (formerly *HER2* or *HER2/neu*).

## Discussion

In this study, we first found that BCSS and OS of patients with only DLNM were not significantly different from those of patients with ISLM. Patients with DLNM showed favorable survival, similar to that of patients with ISLM (stage IIIC disease) and better than that of patients with stage IV breast cancer (DLNM excluded). Moreover, locoregional treatment was associated with improved survival for DLNM cases.

Clinical metastases of malignant tumors are organ specific.^8^ This selective metastatic process relies on highly sophisticated and complex interactions between the metastatic cell surface and the capillary endothelium in various recipient organs^17,18^ or the stroma or high endothelial venule of the lymph node.^19^ Lymph node metastases themselves are nonlethal, but they predict poor patient survival. Previous studies^7,20,21^ suggest that contralateral lymph node metastases show favorable clinical outcomes, appearing better than those at other sites of distant metastases. In the current study, our results suggest that patients with ISLM and DLNM were associated with improved BCSS and OS when compared with patients with distant metastases (DLNM excluded). All previously mentioned data suggest that breast cancer lymph node metastases were different than other distant sites of metastases.

Stage IV breast cancer remains a virtually incurable disease, with the main goals of treatment being prolongation of survival and palliation of symptoms.^3,22^ Systemic therapy, including chemotherapy, endocrine therapy, and anti-*ERBB2* therapy, is the main treatment for stage IV breast cancer. Stage I to III breast cancer is a curable disease, and locoregional treatment with or without systemic therapy is the main therapy. In the present cohort study, we found that patients with DLNM showed comparable survival with patients who had ISLM in any subtype. Patients with DLNM may have a curable disease in the current situation, and this disease may be classified as an N3 disease, not a stage IV disease.

For stage IV breast cancer, the role of locoregional surgery is controversial, and locoregional surgery has been shown to have a significant detrimental association with distant progression-free survival,^2,3^ especially for visceral metastases. In the current study, 55.8% of patients with DLNM received surgery for the primary tumor, which was associated with improved survival in all molecular subtypes. Additionally, 36.7% of these patients received radiotherapy, which was also associated with improved survival for some subtypes in this study. Therefore, we recommend aggressive radical locoregional therapy for patients with DLNM to improve their survival in the future.

### Limitations

Several limitations exist in the current study. First, the treatment principles of stage III and stage IV breast cancer are different, so the survival of patients with DLNM and ISLM may be influenced by different therapies. However, our results represent the real-world survival of these patients. Second, several important clinical characteristics and treatment data were not available in the current study.^23^ Last, because of the study’s retrospective design, the limitations of the database,^24,25,26^ and the relatively short follow-up, prospective studies with long-term follow-up are needed to confirm our results.

## Conclusions

This cohort study is the first, to our knowledge, to give direct evidence from a large sample size that breast cancer with DLNM may be a curable disease, similar to N3c disease, with a significantly better prognosis than other types of stage IV disease (DLNM excluded). Locoregional treatment was associated with significantly improved survival for patients with DLNM. Our findings suggest that DLNM of breast cancer may be a regional disease, not a metastatic disease, and it is necessary to reassess the role of lymph node metastases of breast cancer.

## Notes

Supplement. **eTable 1.** Three Groups of Patients Included in the Cohort Study **eTable 2.** Univariate Analysis for Breast Cancer–Specific Survival and Overall Survival for all Included Patients **eTable 3.** Characteristics of Patients in ISLM Group and DLNM Group After Propensity Score Matching **eTable 4.** Treatment Information of the Three Groups **eTable 5.** Univariate Analysis for Breast Cancer–Specific Survival and Overall Survival for Patients With Distant Lymph Node Metastases **eFigure.** Subgroup Analysis of Primary Surgery and Radiotherapy in Breast Cancer Patients With DLNM
