# Supplementary material for: Comparison of Survival Outcomes Among Patients With Breast Cancer With Distant vs Ipsilateral Supraclavicular Lymph Node Metastases
Source: JAMA Netw Open. 2021 Mar 16;4(3):e211809. doi: 10.1001/jamanetworkopen.2021.1809 (PMC7967083; doi:10.1001/jamanetworkopen.2021.1809)
Supplement: Supplement. — eTable 1. Three Groups of Patients Included in the Cohort Study eTable 2. Univariate Analysis for Breast Cancer–Specific Survival and Overall Survival for all Included Patients eTable 3. Characteristics of Patients in ISLM Group and DLNM Group After Propensity Score Matching eTable 4. Treatment Information of the Three Groups eTable 5. Univariate Analysis for Breast Cancer–Specific Survival and Overall Survival for Patients With Distant Lymph Node Metastases eFigure. Subgroup Analysis of Primary Surgery and Radiotherapy in Breast Cancer Patients With DLNM [file jamanetwopen-e211809-s001.pdf]

Supplementary Online Content

Pan H, Wang H, Qian M, et al. Comparison of survival outcomes among patients with breast cancer with distant vs ipsilateral supraclavicular lymph node metastases. *JAMA Netw Open*. 2021;4(3):e211809. doi:10.1001/jamanetworkopen.2021.1809

eTable 1. Three Groups of Patients Included in the Cohort Study

eTable 2. Univariate Analysis for Breast Cancer–Specific Survival and Overall Survival for all Included Patients

eTable 3. Characteristics of Patients in ISLM Group and DLNM Group After Propensity Score Matching

eTable 4. Treatment Information of the Three Groups

eTable 5. Univariate Analysis for Breast Cancer–Specific Survival and Overall Survival for Patients With Distant Lymph Node Metastases

eFigure. Subgroup Analysis of Primary Surgery and Radiotherapy in Breast Cancer Patients With DLNM

This supplementary material has been provided by the authors to give readers additional information about their work.

© 2021 Pan H et al. *JAMA Network Open.*

eTable 1. Three Groups of Patients Included in the Cohort Study

Groups	Definition	Tumor size	Axillary lymph node stage (N0-N3b)	ISLM (N3c)	DNLM only	Distant metastasis (only DLNM excluded)
**ISLM group**	Patients with ipsilateral supraclavicular lymph node metastases (ISLM)	Any	Any	Yes	No	No
**DNLM group**	Patients with only distant lymph node metastases (DNLM), which considered as stage IV diseases according to AJCC classification. Distant lymph nodes include cervical, contralateral/bilateral axillary and/or internal mammary, transverse cervical and other distant nodes metastases than above	Any	Any	Any	Yes	No
**Distant metastasis group**	Patients with distant metastases (only DLNM excluded)	Any	Any	Any	No	Yes

© 2021 Pan H et al. *JAMA Network Open.*

eTable 2. Univariate Analysis for Breast Cancer–Specific Survival and Overall Survival for all Included Patients

**Variable**	**Breast cancer-specific survival**			**Overall survival**		
	**HR**	**95%CI**	***P* value**	**HR**	**95%CI**	***P* value**
**Age**						
≤50	Reference			Reference		
>50	1.84	1.49-2.27	< .001	1.92	1.59-2.31	< .001
**Race**						
White	Reference			Reference		
Black	1.21	0.99-1.49	.065	1.19	1.01-1.41	.043
Other	0.93	0.69-1.24	.607	0.97	0.77-1.22	.794
**Histology**						
IDC	Reference			Reference		
ILC	1.49	1.16-1.93	.002	1.31	1.05-1.62	.015
Other	1.80	1.51-2.15	< .001	1.75	1.52-2.02	< .001
**Grade**						
Well	Reference			Reference		
Moderately	0.91	0.61-1.37	.664	0.98	0.71-1.35	.908
Poorly/undifferentiated	1.11	0.75-1.65	.587	1.19	0.88-1.62	.268
**Subtypes**						

© 2021 Pan H et al. *JAMA Network Open.*

HR+/HER2-	Reference			Reference		
HR+/HER2+	0.59	0.44-0.79	< .001	0.65	0.51-0.82	< .001
HR-/HER2+	0.86	0.65-1.16	.326	0.89	0.69-1.15	.371
HR-/HER2-	1.77	1.42-2.20	< .001	1.80	1.49-2.16	< .001
**Stage group**						
ISLM	Reference			Reference		
DLNM	0.93	0.64-1.36	.715	0.81	0.59-1.10	.170
Distant metastases	2.17	1.61-2.92	< .001	1.90	1.49-2.42	< .001

ISLM, ipsilateral supraclavicular lymph node metastases; DLNM, distant lymph node metastases; IDC, invasive ductal carcinoma; ILC, invasive lobular carcinoma

© 2021 Pan H et al. *JAMA Network Open.*

eTable 3. Characteristics of Patients in ISLM Group and DLNM Group After Propensity Score Matching

**Variables**	**ISLM**	**DLNM**	***P* value**
**Total**	**143**	**143**	
**Age**			.208
≤50	52	42	
>50	91	101	
**Race**			.279
White	104	101	
Black	26	24	
Other	9	17	
Unknown	4	1	
**Histology**			.652
IDC	113	119	
ILC	12	9	
Other	18	15	
**Grade**			.896
Well	4	5	
Moderately	39	41	
Poorly/undifferentiated	100	97	
**Subtypes**			.224
HR+/HER2-	60	47	
HR+/HER2+	23	34	
HR-/HER2+	20	25	
HR-/HER2-	40	37	

eTable 4. Treatment Information of the Three Groups

**Variables**	**ISLM**	**DLNM**	**Distant metastasis**	***P* value**
**Total**	**212**	**346**	**1475**	
**Primary surgery**				< .001
No	68	151	1047	
Yes	144	193	411	

© 2021 Pan H et al. *JAMA Network Open.*

Unknown	0	2	17	
**Surgery for metastasis**				< .001
No	208	279	1374	
Yes	2	52	2	
Unknown	2	15	99	
**Radiation therapy**				< .001
No	64	195	1045	
Yes	133	127	401	
Unknown	15	24	29	
**Chemotherapy**				< .001
No	30	109	775	
Yes	182	237	700	
Unknown	0	0	0	

© 2021 Pan H et al. *JAMA Network Open.*

eTable 5. Univariate Analysis for Breast Cancer–Specific Survival and Overall Survival for Patients With Distant Lymph Node Metastases

**Variable**	**Breast cancer-specific survival**				**Overall survival**			
	**Number**	**HR**	**95%CI**	***P* value**	**Number**	**HR**	**95%CI**	***P* value**
**Total**	256				346			
**Age**								
≤50	63	Reference			71	Reference		
>50	193	1.28	0.71-2.30	.414	275	1.61	0.94- 2.77	.084
**Race**								
White	184	Reference			253	Reference		
Black	41	1.08	0.58-2.01	.798	56	1.15	0.70-1.90	.587
Other	28	0.44	0.14-1.40	.164	37	0.61	0.26-1.40	.240
**Histology**								
IDC	179	Reference			236	Reference		
ILC	12	3.10	1.20-7.96	.019	21	1.25	0.50-3.12	.635
Other	65	2.06	1.20-3.54	.009	89	1.52	0.98-2.37	.060
**T stage**								
T1	33				58	Reference		
T2	63	1.81	0.65-5.03	.256	81	1.37	0.63-3.00	.427

© 2021 Pan H et al. *JAMA Network Open.*

T3	33	0.70	0.17-2.93	.624	46	1.10	0.45-2.70	.839
T4	105	2.44	0.94-6.33	.067	129	2.39	1.20-4.76	.013
**N stage**								
N0	21	Reference			48	Reference		
N1	85	1.54	0.53-4.49	.430	110	1.81	0.87-3.78	.112
N2	32	0.36	0.07-1.96	.236	39	0.40	0.11-1.47	.166
N3	110	1.61	0.57-4.57	.372	132	1.63	0.79-3.36	.186
**Grade**								
Well	10	Reference			17	Reference		
Moderately	65	0.45	0.12-1.68	.235	98	0.58	0.21-1.59	.293
Poorly/undifferentiated	133	0.69	0.21-2.27	.545	170	0.95	0.38-2.37	.904
**Subtypes**								
HR+/HER2-	101	Reference			151	Reference		
HR+/HER2+	50	0.57	0.23-1.41	.222	59	1.05	0.54-2.06	.878
HR-/HER2+	32	0.36	0.11-1.20	.096	39	0.67	0.28-1.61	.373
HR-/HER2-	54	2.68	1.47-4.89	.001	70	3.11	1.88-5.15	< .001
**Primary surgery**								
No	120	Reference			151	Reference		
Yes	136	0.22	0.13-0.39	< .001	139	0.27	0.18-0.42	< .001

© 2021 Pan H et al. *JAMA Network Open.*

**Distant surgery**								
No	220	Reference			279	Reference		
Yes	30	0.94	0.40-2.19	.886	52	0.62	0.31-1.24	.178
**Radiotherapy**								
No	131	Reference			195	Reference		
Yes	108	0.32	0.18-0.59	< .001	127	0.39	0.24-0.64	< .001

© 2021 Pan H et al. *JAMA Network Open.*

eFigure. Subgroup Analysis of Primary Surgery and Radiotherapy in Breast Cancer Patients With DLNM

	HR(95% CI)	*P*
**BCSS**		
Surgery for primary tumor		
Subtypes		
HR+/HER2−	0.18(0.06−0.48)	.001
HER2+	0.41(0.11−1.53)	.183
HR−/HER2−	0.09(0.03−0.27)	<.001
Radiotherapy		
Subtypes		
HR+/HER2−	0.17(0.05−0.59)	.005
HER2+	0.32(0.07−1.61)	.168
HR−/HER2−	0.36(0.16−0.83)	.016
**OS**		
Surgery for primary tumor		
Subtypes		
HR+/HER2−	0.30(0.14−0.62)	.001
HER2+	0.34(0.13−0.89)	.028
HR−/HER2−	0.09(0.03−0.21)	<.001
Radiotherapy		
Subtypes		
HR+/HER2−	0.35(0.14−0.86)	.021
HER2+	0.28(0.08−0.97)	.044
HR−/HER2−	0.36(0.16−0.83)	.016

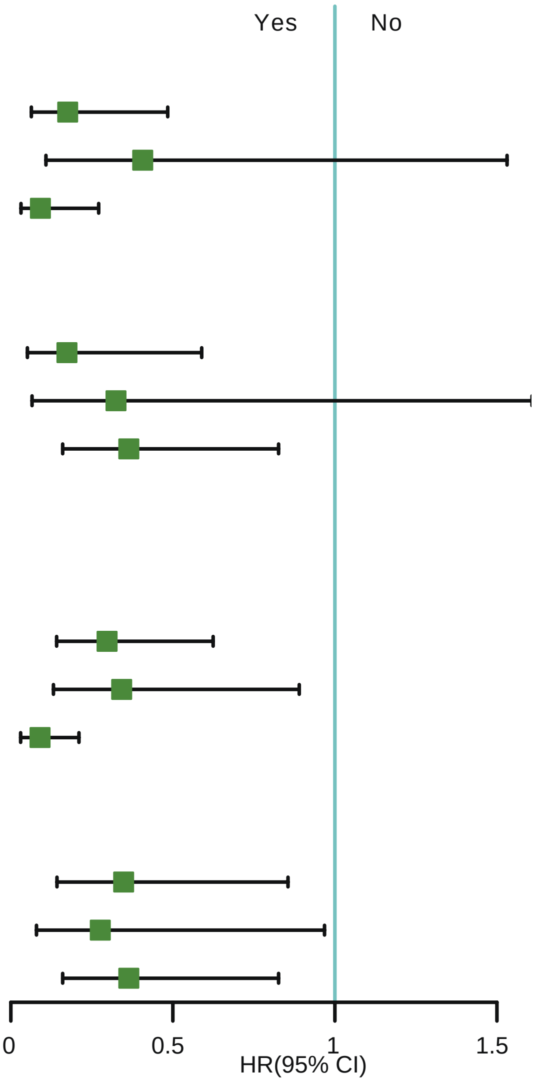

© 2021 Pan H et al. *JAMA Network Open.*
